# Hidden SARS-CoV-2 Omicron Infections in Young Children: What Routine Tests Do Not Tell

**DOI:** 10.1055/s-0045-1809649

**Published:** 2026-03-03

**Authors:** Stijn Bogaert, Hannah Lukasik, Panagiotis Georgiou, Philipp Gude, Sonja Farajzadeh, Stefan Dazert, Olaf Michel, Konstantin van Ackeren, Stefan Volkenstein

**Affiliations:** 1 Department of Otorhinolaryngology – Head and Neck Surgery, Saint Elisabeth Hospital, Ruhr University Bochum, Bochum, Nordrhein-Westfalen, Germany; 2 Department of Otorhinolaryngology – Head and Neck Surgery, Johannes Wesling Hospital Minden, Johannes Wesling Medical Center Minden, Ruhr University Bochum, Minden, Nordrhein-Westfalen, Germany; 3 Department of Otorhinolaryngology, Head and Neck Surgery, Larnaka General Hospital, European University Cyprus, Larnaka, Cyprus; 4 Department of Anesthesiology, Saint Elisabeth Hospital, Ruhr University Bochum, Bochum, Nordrhein-Westfalen, Germany; 5 Department of Otorhinolaryngology – Head and Neck Surgery, University Hospital Brussels, Vrije Universiteit Brussel, Brussels, Belgium; 6 Department of Ear, Nose, and Throat (ENT), ENT Center Middle Hesse, Marburg, Germany

**Keywords:** COVID-19 testing, pediatrics, predictive value of tests, clinical practice guideline, lymphoid tissue

## Abstract

**Introduction:**

The coronavirus disease 2019 (COVID-19) pandemic caused a global emergency. Screening protocols vary regarding the epidemiological situation and the dominant virus variant. Implementing these protocols can be particularly challenging in young children.

**Objective:**

To evaluate the diagnostic accuracy of routine polymerase chain reaction (PCR) testing in small children. Furthermore, hidden severe acute respiratory syndrome coronavirus-2 (SARS-CoV-2) Omicron infections, missed by routine PCR testing, were intraoperatively analyzed, and multiple testing methods were compared.

**Methods:**

The present prospective cohort study was performed between March and May 2022. All children aged ≤ 6 years who were admitted for adenoidectomy and/or tonsillotomy to the Department of Otorhinolaryngology – Head and Neck Surgery of the Saint Elisabeth Hospital, in Bochum, Germany, were included. Routine PCR swabs were performed ≤ 24 hours before surgery. Intraoperatively, rapid antigen tests, separate naso- and oropharyngeal PCR swabs, adenoid and/or tonsillar tissue for PCR analysis, and serological tests were collected.

**Results:**

We included 55 children with negative preoperative PCR tests. Intraoperatively, SARS-CoV-2 particles were detected in 51% of the sample. Among children without a history of SARS-CoV-2 infection within 90 days before surgery, the prevalence was 43%. Compared with the PCR results regarding tissue, the preoperative PCR screening had a sensitivity of 20%, and, in the intraoperative PCR screening, the optimally performed swabs had a sensitivity of 79%. In total, 29% of the positive cases had a cycle threshold (Ct) value < 30. Nasopharyngeal PCR tests detected significantly more SARS-CoV-2 infections than oropharyngeal swabs.

**Conclusion:**

Routine PCR tests in infants may present a high rate of false-negative results and a low sensitivity. These findings question preoperative screening protocols that include testing asymptomatic children who have recovered from a recent SARS-CoV-2 infection.

## Introduction

Since 2019, severe acute respiratory syndrome coronavirus-2 (SARS-CoV-2) has caused a pandemic that continues to influence the medical landscape worldwide. Currently, the Omicron variant of concern (VoC) is still the primary driver of the pandemic and is highly infectious, leading to peak incidence rates. 1 In most hospitals, elective surgeries were postponed preventing virus transmission, especially in otorhinolaryngology, as the upper respiratory tract is the port of entry and harbor of SARS-CoV-2. Screening protocols varied regarding the epidemiological situation and the dominant virus variant. One of the measures to ensure complication-free anesthesia for patients and a safe environment for healthcare personnel was to perform preoperative SARS-CoV-2 polymerase chain reaction (PCR) tests strictly on all elective patients. The latest guideline by the Centers for Disease Control and Prevention (CDC), the national public health agency of the United States, does not recommend testing for asymptomatic people who have recovered from SARS-CoV-2 infection within the previous 90 days. 2 Several coronavirus disease 2019 (COVID-19) measures have been lifted, but they may be reinstated if a new highly-pathogenic variant emerges. 3 Hospital infection protection measures and testing are still essential in sensitive areas. The recognized standard is the rapid antigen diagnostic (RAD) test of SARS-CoV-2 or the reverse transcriptase-PCR (RT-PCR) smear of the oro- and nasopharynx.

In young children, infection control measures are challenging. Performing an optimal nasopharyngeal swab (NPS) or oropharyngeal swab (OPS) is difficult due to discomfort and consequent low compliance. Studies 4 5 have reported a traumatizing impact of testing on children, inversely related to age. Infections by SARS-CoV-2 in children are underdiagnosed, and infections in children by the Omicron variant are more frequently asymptomatic than infections by earlier variants. 1 6 7 Younger children present a higher rate of Omicron infection and are more likely to have severe Omicron COVID-19 than older children. 8 9 10 11 Moreover, the relative risk of COVID-19-related hospitalization and Intensive Care Unit (ICU) admission decreased during the Omicron period, except for children under 5 years of age. 12 13 Hidden SARS-CoV-2 infections are of no concern in otologic surgery. 14 The highest viral load, however, is found in the nasopharynx of both symptomatic and asymptomatic children with SARS-CoV-2 infections. 15 Asymptomatic children can also exhibit a high viral load, indicating the existence of hidden SARS-CoV-2 infections in the nasopharynx. 16

The present study aimed to evaluate the diagnostic accuracy of routine PCR tests in young children and to determine the prevalence of hidden SARS-CoV-2 Omicron infections, missed by routine PCR testing. Therefore, routine PCR tests were compared with the results of standard SARS-CoV-2 diagnostic tests collected intraoperatively and PCR results of adenoid and/or tonsillar tissue samples.

## Methods

### Design, Population, and Sampling

The present prospective cross-sectional study was conducted after approval from the institutional Review Board (under registration number 22–7465) and was registered in the German Clinical Trials Register (DRKS00029030). The study was performed in accordance with the principles of the 2000 revision of the Declaration of Helsinki and the European Medicines Agency (EMA) Guidelines for Good Clinical Practice (2017). The a-priori calculation using the G*Power software, version 3.1 (free) suggested a minimum sample size of 54 to achieve a statistical power of 80% and a type-I error rate < 0.05. 17 18 The raw dataset is available in a public repository. 19

Children aged ≤ 6 years who were admitted for routine adenoidectomy and/or tonsillotomy to the Department of Otorhinolaryngology – Head and Neck Surgery of the Saint Elisabeth Hospital, in Bochum, Germany, were prospectively recruited through consecutive sampling between March and May 2022.

A total of 67 patients were eligible to participate in the study. The legal guardians of 62 of them agreed to participate and gave their informed consent. After excluding 7 children due to a positive SARS-CoV-2 PCR swab result ≤ 24 hours preoperatively, 55 patients were finally available for further investigation. The treatment medical staff wore at least an FFP2 mask and eye protection.

### RT-PCR Swabs

A PCR swab was routinely performed ≤ 24 hours preoperatively in all patients by medically trained personnel. The swab was rubbed over the posterior wall of the oro- and nasopharynx with a slight twisting motion for ∼ 10 seconds or as long as the patient was compliant.

After induction of general anesthesia, the surgeon brushed one OPS over the posterior and lateral walls of the oropharynx and another NPS over the nasopharynx under visual control for ∼ 10 seconds. The smears were sent to the central laboratory of the hospital for processing within 2 hours. The SARS-CoV-2 RNA and the analysis of the cycle threshold (Ct) value were tested using the Allplex SARS-CoV-2 Fast PCR Assay (Seegene) following the manufacturer's instructions. 20

### RAD

Intraoperatively, the surgeon rubbed each swab on the posterior wall of the oropharynx and in the nasopharynx for ∼ 10 seconds. The commercially available antigen test kit (Green Spring SARS-CoV-2 Antigen Rapid Test Kit, Colloidal Gold, Shenzhen Lvshiyuan Biotechnology Co., Ltd.) was used. 18 21 The RADs were performed and evaluated according to the manufacturer's guidelines.

### RT-PCR on Tissue Samples

The extraction and PCR methods described in literature were validated in advance in our laboratory. 22 After adenoidectomy and tonsillotomy, a portion of the tissue was preserved in RNAprotect Tissue Reagent (QIAGEN) by a technical assistant. The tissue was incubated overnight at 4 °C, cut into pieces, and ∼ 0.2 g was stored at -80 °C. The RT-PCR analysis was conducted as a pure research test by the Institute of Medical Laboratory Diagnostics, in Bochum, Germany. The investigator was blinded regarding the clinical data and other results. After homogenization and centrifugation, the supernatant was incubated with proteinase K and lysis buffer (Promega). Total nucleic acid was extracted using the automated Maxwell RSC Blood DNA Kit (Promega). The Allplex SARS-CoV-2 assay (Seegene) was used for the RT-PCR analysis.

### Serological Tests

After the insertion of intravenous access for anesthesia, a blood sample was obtained in a serum tube. The detection of antibodies against SARS-CoV-2 spike protein was performed using the Elecsys Anti-SARS-CoV-2 S immunoassay (Roche Diagnostics International Ltd.,).

### Statistics

All collected data were merged in a Microsoft Excel (Microsoft Corp.) spreadsheet and underwent automated and manual verification. The statistical analysis was performed using the IBM SPSS Statistics for Windows software, version 25.0 (IBM Corp.). Predictive values of the intraoperative RT-PCR swabs were calculated in comparison to the RT-PCR results on tissue using Bayes' theorem, based a point prevalence of 1.4% in the general population. 23 Positive likelihood ratios could not be calculated because of the 100% sensitivity of all PCR tests. The appropriate variables underwent statistical analysis using the Spearman's rank correlation coefficient, Chi-squared test, Mann-Whitney U-test, Student's *T* -test, and the Kruskal Wallis one-way analysis of variance.

## Results

Between March and May 2022, 55 patients with negative preoperative RT-PCR screening were included in the study. Their mean age was of 3.5 (standard deviation: ± 1.08) years, and there were 25 (46%) female and 30 (54%) male subjects. The demographic and clinical characteristics are listed in Table 1 . Adenoid tissue was collected in 47 (86%) cases and tonsillar tissue, in 7 (13%).

**Table 1:** Patient characteristics

Variables		Range
Age (years): mean(± SD)	3.5(± 1.08)	(1.4–5.8)
Female sex: n (%)	25 (46)	
Proven previous SARS-CoV-2 infection ( *n* = 53): n (%)	9 (17)	
Interval before surgery (days): mean(± SD)	82.4(± 106.61)	(23–362)
Adenoid tissue collection: n (%)	47 (86)	
Tonsillar tissue collection: n (%)	7 (13)	
Previous COVID-19 vaccination: n (%)	1 (2)	

Abbreviations: COVID-19, coronavirus disease 2019; SARS-CoV-2, severe acute respiratory syndrome coronavirus-2; SD, standard deviation.

Severe acute respiratory syndrome coronavirus-2 RNA was detected in 49% of the adenoids and in 14% of the tonsil tissue. The intraoperative RT-PCR swabs were positive in 35% when collected in the nasopharynx and in 12% in the oropharynx. Table 2 provides a detailed overview of the RT-PCR results. Overall, this equals the prevalence of SARS-CoV-2 infection of 51% of all included children. A Ct value < 30 was detected in 8 children, which constituted 29% of the positive results. In all cases with proven previous SARS-CoV-2 infection, the PCR was still positive (mean Ct value: 30.2). Particles of SARS-CoV-2 were also detected in 43% of children without a history of SARS-CoV-2 infection 90 days before surgery. The NPS detected significantly more SARS-CoV-2 infections than the OPS (odds ratio [OR] = 14.2, 95%CI: 1.5%–133.8%; *p* = 0.002). The combination of NPS and OPS showed SARS-CoV-2 particles in 37% of all patients, corresponding to a sensitivity of 74%. The RAD was negative in all patients. A previous SARS-CoV-2 infection was known in 32% of the positive cases, and the mean interval since the first diagnosis was of 82.4 days before surgery. Table 2 provides a detailed overview of the RT-PCR results.

**Table 2:** RT-PCR diagnostics for SARS-CoV-2

Test	Positive: n (%)	Ct: mean(± SD)
NPS ( *n* = 54)	19 (35)	33.0(± 3.22)
OPS ( *n* = 52)	6 (12)	35.5(± 2.94)
Adenoid ( *n* = 47)	23 (49)	32.8 ± 2.96)
Tonsil ( *n* = 7)	1 (14)	32.0

**Abbreviations:** Ct, cycle threshold; NPS, nasopharyngeal polymerase chain reaction swab; OPS, oropharyngeal polymerase chain reaction swab; RT-PCR, reverse transcriptase-polymerase chain reaction; SARS-CoV-2 **,** severe acute respiratory syndrome coronavirus-2 **;** SD, standard deviation.

The PCR test on adenoid tissue detected the most positive cases. All patients with positive adenoids and a Ct value < 30 were also detected through the NPS. Conversely, all patients with a positive NPS had positive viral RNA detection in the tissue samples of the adenoids. The negative predictive value (NPV), the positive predictive value (PPV), the sensitivity, and the specificity of the RT-PCR swabs compared with the PCR results on tissue are listed in Table 3 . When serum samples were available ( *n* = 42), antibodies against SARS-CoV-2 were found in 18 (43%) patients. In 7 (30%) of the cases with a positive PCR test, no antibodies were found. In this subgroup with positive PCR test and a negative serology, the mean Ct value was 34.1.

**Table 3:** Validation of intraoperative PCR swabs

RT-PCR test	NPV (%)	PPV (%)	Sensitivity (%)	Specificity (%)	NLR
NPS + OPS	99.6	100.0	74.1	100.0	0.26
NPS	99.6	100.0	70.4	100.0	0.30
OPS	98.9	100.0	24.0	100.0	0.76

**Abbreviations:** Ct, cycle threshold; NLR, negative likelihood ratio; NPS, nasopharyngeal polymerase chain reaction swab; NPV, negative predictive value; OPS, oropharyngeal polymerase chain reaction swab; PPV, positive predictive value; RT-PCR, reverse transcriptase-polymerase chain reaction.

Seven cases could not be included in the previous analyses because of positive preoperative SARS-CoV-2 PCR results. This means that the preoperative PCR swab reached an NPV of 47%, a sensitivity of 20%, and a negative likelihood ratio of 0.8 ( Supplementary Table S1 ; online only). Combining the pre- and intraoperative PCR results, a sensitivity of 79% could be achieved.

## Discussion

In the current prospective cross-sectional study of children aged ≤ 6 years undergoing adenoidectomy with or without tonsillotomy, SARS-CoV-2 particles were detected in half of the patients, despite having negative preoperative PCR tests from the naso- and oropharynx. Among the positive cases, 29% had Ct values < 30, indicating a high viral load. Intraoperative NPS diagnosed all these active infections. The preoperative PCR screening had a sensitivity of only 20%. In contrast, optimally performed NPS/OPS had a sensitivity of 79%. These results suggest that PCR testing may not be reliable in awake children with the Omicron variant, raising questions regarding the preoperative screening protocol for this specific patient group. These findings contrast with those of a previous study conducted early in 2021 by Osterbauer et al., 22 who found that 19% of children aged ≤ 16 years with negative preoperative NPS had virus particles in adenoid tissue, as detected by RT-PCR.

In the present study, only 32% of the positive cases had a history of SARS-CoV-2 infection. This supports previous studies 1 7 that suggest that the incidence of SARS-CoV-2 infections in children is underestimated. In the current study, in children without a prior SARS-CoV-2 infection 90 days before surgery, hidden virus particles were detected in 43% of the cases. It has been shown 24 that long-term PCR-positive patients can carry infectious viruses. The duration of viable virus shedding for the SARS-CoV-2 Omicron variant is of 5 days, and the average duration of PCR positivity is of 11 days. 25 These durations are similar in both symptomatic and asymptomatic patients, but there is high variability, and younger age is associated with prolonged viral shedding. 25 26 27 In the current study, virus particles were still detected in all children with a known history of SARS-CoV-2 infection. The mean time between the first diagnosis and surgery was of 82.4 (range: 23–362) days, and the mean Ct value was of 30.2. These findings suggest that preoperative screening protocols that include routine preoperative PCR testing for all patients may not be necessary and support the current guidelines of the CDC, which do not recommend testing for asymptomatic people who have recovered from SARS-CoV-2 infection within the previous 90 days. 2 28

Positive evolutionary selection has led to the development of more than 30 mutations in the SARS-CoV-2 spike protein, resulting in reduced sensitivity of conventional RT-PCR and RAD. 29 The current study confirms that NPS has significantly higher sensitivity than OPS in detecting the Omicron variant in children. 30 31 32 Therefore, relying solely on an OPS in children is inadequate. However, RT-PCR is unable to differentiate between infectious and non-infectious patients. The Ct value provides a better, albeit relative and semiquantitative, measure of infectiousness. 33

The current study provides further insight into the circulation of the SARS-CoV-2 Omicron variant in children aged ≤ 6 years. Medical staff are at an increased risk of exposure to the virus through children with undetected SARS-CoV-2 infections, emphasizing the need to wear personal protective equipment during invasive procedures.

Intraoperative OPS/NPS PCR swabs are an efficient method of detecting children with false-negative SARS-CoV-2 results, and they may be a useful technique for future coronavirus variants. However, it takes several hours to process a PCR swab, and the result of an intraoperative PCR test can only be known after surgery. RAD could solve this problem, but, despite its optimal performance in the current study, the result was negative in all cases, including 8 children with SARS-CoV-2 infections with a Ct value < 30. This finding suggests a low sensitivity of current RAD test kits in children aged ≤ 6 years, and our results indicate that RAD is not reliable in detecting SARS-CoV-2 with the Omicron variant in young children. Previous studies 34 in the general population have reported reduced detection rates of Omicron infections with the current RAD. Note that RAD sensitivity is typically calculated in comparison to PCR swabs, which, in turn, are not 100% sensitive, as shown in the present study. Point-of-care tests (POCTs) with nucleic acid amplification techniques (NAATs) have a high potential for rapid and sensitive diagnostics, but their use is limited by their high costs and variable sensitivity. 35

Children may be less likely than adults to have seroconversion despite similar viral loads. 36 About 24 to 37% of SARS-CoV-2-positive children fail to seroconvert. 26 36 Omicron-infected children mount a significantly lower antibody response than those infected by pre-Omicron variants. 37 During the period herein analyzed, vaccination was not yet recommended for children in Germany. 38 Indeed, almost one third of the patients with a positive PCR did not have antibodies in the current study. This proves that serological tests in young children have a heterogenous sensitivity in the diagnosis of SARS-CoV-2 infections and confirms that the seroconversion rate in children is limited. 26 36 Since all children with known prior SARS-CoV-2 infection still had virus particles in lymphoid tissue, the PCR test on the adenoids might provide a better idea of the incidence in young children than the serological test. The collection of serum samples at a later time was not intended in the current study, so the seroconversion rate in our sample is unclear. The introduction of vaccination programs for children may complicate the interpretation of serological test results but did not influence our study.

The current is the first study that validates both RAD and PCR swabs compared with lymphoid tissue for the diagnosis of the Omicron variant. We exclusively included children under the age of 6 years, which is a unique patient group in this context because of the low compliance in nasopharyngeal testing, difficulties in infection protection measures, and an altered vaccination schedule. All preoperative PCR tests in the present study were performed within a narrow time frame of 24 hours before the intraoperative diagnostics. General anesthesia enabled the collection of intraoperative swabs according to the standard and, thereby, bypassing the low compliance in infants and excluding preanalytical biases. 39

Th current study has a few limitations. Children with a positive preoperative PCR test and thus possibly highly infectious disease did not receive further diagnostics within the scope of the study. Secondly, serologic testing was only performed in 42 (76%) of the patients, which did not enable us to draw conclusions about the stage of the detected infections. The sample size is limited due to the intentions of this cohort study. Based on the results presented, additional larger studies can be initiated to optimize screening protocols in the current phase of the pandemic. Further mapping of the virus particles in the nasal cavity, particularly a comparison with the anterior portion of the inferior turbinate, might optimize the brushing technique in children.

## Conclusion

The current study demonstrates the limitations of routine preoperative PCR swabs to detect SARS-CoV-2 in infants. Routine PCR tests in this patient group may have a high rate of false-negative results and a low sensitivity. Despite testing negative on preoperative PCR tests, nearly half of children aged ≤ 6 years were found to have SARS-CoV-2 virus particles during surgery, indicating a hidden SARS-CoV-2 Omicron infection. In almost 30% of these cases, the Ct value was < 30. The present study confirms that the sensitivity of NPSs is significantly higher than that of OPSs for the Omicron variant in children. Additionally, our results question the reliability of RAD in this patient group. All 7 patients with previous SARS-CoV-2 infections tested positive in the RT-PCR analysis, with a mean interval from diagnosis of 82 days prior to the surgery. These findings challenge preoperative screening protocols that include testing asymptomatic children who have recently recovered from SARS-CoV-2 infections.

## References

[1] Clarke K EN Jones J M Deng Y Nycz E Lee A Iachan R Seroprevalence of Infection-Induced SARS-CoV-2 Antibodies - United States, September 2021-February 2022 MMWR Morb Mortal Wkly Rep 2022 71 17 606 608 10.15585/mmwr.mm7117e3 35482574 PMC9098232

[2] CDC Overview of Testing for SARS-CoV-2, the virus that causes COVID-19 2023Accessed August 31, 2024 from:https://archive.cdc.gov/#/details?url=https://www.cdc.gov/coronavirus/2019-ncov/hcp/testing-overview.html

[3] Otake T Government to scale back COVID support with disease downgrade on May 8The Japan Times2023Available from:https://www.japantimes.co.jp/news/2023/03/10/national/scale-back-covid-support/

[4] LAVA study team Harwood R Rad L Larru B Kelly C Kenny S Comparison of the pain experienced with anterior nasal swabs and nose and throat swabs in children Arch Dis Child 2022 107 02 207 10.1136/archdischild-2021-321708 34728461 PMC8785051

[5] Slifer K J Tucker C L Dahlquist L M Helping Children and Caregivers Cope with Repeated Invasive Procedures: How Are We Doing? J Clin Psychol Med Settings 2002 9 131 152 10.1023/A:1014944110697

[6] Smith H Mahon A Moss A Rao S SARS-CoV-2 infection in children evaluated in an ambulatory setting during Delta and Omicron time periods J Med Virol 2023 95 01 e28318 10.1002/jmv.28318 36397139

[7] Couture A Lyons B C Mehrotra M L Sosa L Ezike N Ahmed F S, et al. Severe Acute Respiratory Syndrome Coronavirus 2 Seroprevalence and Reported Coronavirus Disease 2019 Cases in US Children, August 2020–May 2021. Open Forum Infect Dis 2022;9(3):ofac044 10.1093/ofid/ofac044 PubMed 10.1093/ofid/ofac044 PMC8860150 35198651

[8] Butt A A Dargham S R Loka S Shaik R M Chemaitelly H Tang P Coronavirus Disease 2019 Disease Severity in Children Infected With the Omicron Variant Clin Infect Dis 2022 75 01 e361 e367 10.1093/cid/ciac275 35404391 PMC9047187

[9] Elliott P Bodinier B Eales O Wang H Haw D Elliott J Rapid increase in Omicron infections in England during December 2021: REACT-1 study Science 2022 375 6587 1406 1411 35133177 10.1126/science.abn8347 PMC8939772

[10] Tagarro A Coya O-N Pérez-Villena A Iglesias B Navas A Aguilera-Alonso D Moraleda C Features of COVID-19 in Children During the Omicron Wave Compared With Previous Waves in Madrid, Spain Pediatr Infect Dis J 2022 41 05 e249 e251 10.1097/INF.0000000000003482 PMC8997014 35333818

[11] Chun J Y Jeong H Kim Y Identifying susceptibility of children and adolescents to the Omicron variant (B.1.1.529) BMC Med 2022 20 01 451 10.1186/s12916-022-02655-z 36419108 PMC9684890

[12] Jank M Oechsle A-L Armann J Behrends U Berner R Chao C-M Comparing SARS-CoV-2 variants among children and adolescents in Germany: relative risk of COVID-19-related hospitalization, ICU admission and mortality Infection 2023 51 05 1357 1367 10.1007/s15010-023-01996-y 36787015 PMC9925936

[13] Belay E D Godfred-Cato S SARS-CoV-2 spread and hospitalisations in paediatric patients during the omicron surge Lancet Child Adolesc Health 2022 6 05 280 281 10.1016/S2352-4642(22)00060-8 35189084 PMC8856665

[14] Yamazaki H Yamamoto N Sonoyama T Maruoka H Nasu S Makino A A multicenter study to investigate the positive rate of SARS-CoV-2 in middle ear and mastoid specimens from otologic surgery patients Auris Nasus Larynx 2023 50 02 285 291 10.1016/j.anl.2022.07.007 35945108 PMC9334977

[15] Kam K Q Thoon K C Maiwald M Chong C Y Soong H Y Loo L H SARS-CoV-2 viral RNA load dynamics in the nasopharynx of infected children Epidemiol Infect 2021 149 e18 10.1017/S095026882100008X 33427152 PMC7847743

[16] Yonker L M Boucau J Regan J Choudhary M C Burns M D Young N Virologic features of severe acute respiratory syndrome coronavirus 2 infection in children J Infect Dis 2021 224 11 1821 1829 10.1093/infdis/jiab509 34647601 PMC8643403

[17] Faul F Erdfelder E Buchner A Lang A-G Statistical power analyses using G*Power 3.1: tests for correlation and regression analyses Behav Res Methods 2009 41 04 1149 1160 10.3758/BRM.41.4.1149 19897823

[18] Scheiblauer H Filomena A Nitsche A Puyskens A Corman V M Drosten C Comparative sensitivity evaluation for 122 CE-marked rapid diagnostic tests for SARS-CoV-2 antigen, Germany, September 2020 to April 2021 Euro Surveill 2021 26 44 2.100441E6 10.2807/1560-7917.ES.2021.26.44.2100441 PMC8569926 34738515

[19] Bogaert S Klein H Georgiu P Hidden SARS-CoV-2 Omicron infections in young children Mendeley Data 2022 10.17632/dzzdfn7h6s.1 PMC12956413 41783032

[20] Liotti F M Menchinelli G Marchetti S Morandotti G A Sanguinetti M Posteraro B Cattani P Evaluation of three commercial assays for SARS-CoV-2 molecular detection in upper respiratory tract samples Eur J Clin Microbiol Infect Dis 2021 40 02 269 277 10.1007/s10096-020-04025-0 32885293 PMC7471581

[21] Borro M Salerno G Montori A Petrucca A Anibaldi P Marcolongo A, et al. SARS-CoV-2 Transmission Control Measures in the Emergency Department: The Role of Rapid Antigenic Testing in Asymptomatic Subjects. Healthcare (Basel) 2022;10(5):790 10.3390/healthcare10050790 PubMed 10.3390/healthcare10050790 PMC9140606 35627926

[22] Osterbauer B Yalamanchili R Hochstim C Ge M Bard J D Ference E H Gomez G Preoperative SARS-CoV-2 Screening Fails to Detect Viral Particles Prior to Airway Surgery Laryngoscope 2022 132 08 1665 1667 10.1002/lary.29906 34643283 PMC8661649

[23] Groenheit R Bacchus P Galanis I Sondén K Bujila I Efimova T High prevalence of SARS-CoV-2 Omicron infection despite high seroprevalence, Sweden, 2022 Emerg Infect Dis 2023 29 06 1240 1243 10.3201/eid2906.221862 37141616 PMC10202879

[24] Zahn T Mhedhbi I Hein S Raupach J Miskey C Husria Y Persistence of infectious SARS-CoV-2 particles for up to 37 days in patients with mild COVID-19 Allergy 2022 77 07 2053 2066 10.1111/all.15138 34637150 PMC8652783

[25] Wu Y Guo Z Yuan J Cao G Wang Y Gao P Duration of viable virus shedding and PCR positivity of the SARS-CoV-2 Omicron variant in upper respiratory tract: a systematic review and meta-analysis Int J Infect Dis 2023 129 228 235 10.1016/j.ijid.2023.02.011 36804640 PMC9937726

[26] EPICO-AEP Working Group Tagarro A Sanz-Santaeufemia F J Grasa C Cobos E Yebra J Alonso-Cadenas J A Dynamics of Reverse Transcription-Polymerase Chain Reaction and Serologic Test Results in Children with SARS-CoV-2 Infection J Pediatr 2022 241 126 132000 10.1016/j.jpeds.2021.09.029 34571020 PMC8463102

[27] Liu Y Xu L Piao X Li H Shi L Huang Y Epidemiological, clinical, and household transmission characteristics of children and adolescents infected with SARS-CoV-2 Omicron variant in Shanghai, China: a retrospective, multicenter observational study Int J Infect Dis 2023 129 1 9 10.1016/j.ijid.2023.01.030 36724865 PMC9884399

[28] CDC . Interim Infection Prevention and Control Recommendations for Healthcare Personnel During the Coronavirus Disease 2019 (COVID-19) Pandemic 2022. Accessed February 20, 2023 from:https://www.cdc.gov/coronavirus/2019-ncov/hcp/infection-control-recommendations.html

[29] Le T TB Vasanthakumaran T Hien H NT Hung I-C Luu M N Khan Z A SARS-CoV-2 Omicron and its current known unknowns: A narrative review Rev Med Virol 2023 33 01 e2398 10.1002/rmv.2398 36150052 PMC9538895

[30] Cornette M Decaesteker B Martens G A Vandecandelaere P Jonckheere S From Delta to Omicron SARS-CoV-2 variant: Switch to saliva sampling for higher detection rate J Clin Virol Plus 2022 2 03 100090 10.1016/j.jcvp.2022.100090 35693461 PMC9172253

[31] Chen M Xu J Ying L Cai M Tung T-H Zhou K Clinical practice of rapid antigen tests for SARS-CoV-2 Omicron variant: A single-center study in China Virol Sin 2022 37 06 842 849 10.1016/j.virs.2022.08.008 36049627 PMC9422342

[32] Wikramaratna P S Paton R S Ghafari M Lourenço J Estimating the false-negative test probability of SARS-CoV-2 by RT-PCR Euro Surveill 2020 25 50 2.000568E6 10.2807/1560-7917.ES.2020.25.50.2000568 PMC7812420 33334398

[33] Singanayagam A Patel M Charlett A Bernal J L Saliba V Ellis J Duration of infectiousness and correlation with RT-PCR cycle threshold values in cases of COVID-19, England, January to May 2020 Euro Surveill 2020 25 32 2.001483E6 10.2807/1560-7917.ES.2020.25.32.2001483 PMC7427302 32794447

[34] Osterman A Badell I Basara E Stern M Kriesel F Eletreby M Impaired detection of omicron by SARS-CoV-2 rapid antigen tests Med Microbiol Immunol 2022 211(2-3):105 117 10.1007/s00430-022-00730-z 35187580 PMC8858605

[35] Kang T Lu J Yu T Long Y Liu G Advances in nucleic acid amplification techniques (NAATs): COVID-19 point-of-care diagnostics as an example Biosens Bioelectron 2022 206 114109 10.1016/j.bios.2022.114109 35245867

[36] Toh Z Q Anderson J Mazarakis N Neeland M Higgins R A Rautenbacher K Comparison of seroconversion in children and adults with mild COVID-19 JAMA Netw Open 2022 5 03 e221313 e221313 10.1001/jamanetworkopen.2022.1313 35262717 PMC8908077

[37] Toh Z Q Mazarakis N Nguyen J Higgins R A Anderson J Do L AH Comparison of antibody responses to SARS-CoV-2 variants in Australian children Nat Commun 2022 13 01 7185 10.1038/s41467-022-34983-2 36434068 PMC9700848

[38] Impfkommission (STIKO) S. Beschluss der STIKO zur 17. Aktualisierung der COVID-19-Impfempfehlung. Epidemiol Bull 2022 7 20 10.25646/9538

[39] Torretta S Zuccotti G Cristofaro V Ettori J Solimeno L Battilocchi L Diagnosis of SARS-CoV-2 by RT-PCR using different sample sources: review of the literature Ear Nose Throat J 2021 100 02 131S 138S 10.1177/0145561320953231 32865458 PMC7459180

