# Supplementary material for: Hidden SARS-CoV-2 Omicron Infections in Young Children: What Routine Tests Do Not Tell
Source: Int Arch Otorhinolaryngol. 2026 Mar 3;30(1):1–6. doi: 10.1055/s-0045-1809649 (PMC12956413; doi:10.1055/s-0045-1809649)
Supplement: Supplementary file 1 — Supplementary Material [file 10-1055-s-0045-1809649-s231548.pdf]

Supplementary Table S1 Preoperative RT-PCR swab validation

NPV (%)	PPV (%)	Sensitivity (%)	Specificity (%)	LR–
98.9	100.0	20.0	100.0	0.80

Abbreviations: LR, negative likelihood ratio, NPV, negative predictive value, PPV, positive predictive value.

 © 2025. The Author(s).
